# Resistance training, but not leucine, increased basal muscle protein synthesis and reversed frailty in older women consuming optimized protein intake

**DOI:** 10.1007/s11357-025-01877-2

**Published:** 2025-09-09

**Authors:** Kathryn J. Jacob, Guy Hajj-Boutros, Vita Sonjak, Jean-Phillippe Leduc-Gaudet, Felipe Broering, Charles Robb Flynn, Stéphanie Chevalier, Marie Lamarche, Sabah N. A. Hussain, José A. Morais

**Affiliations:** 1 Research Institute of the McGill University Health Centre, 2155 Guy Street, Suite 500, Montreal, QC H3H 2R9 Canada; 2 Research Group in Cellular Signaling, Department of Medical Biology, Université du Québec À Trois-Rivières, Trois-Rivières, QC G9A 5H7 Canada; 3 Department of Surgery, Vanderbilt University Medical Center, Nashville, TN USA; 4 Division of Geriatric Medicine, McGill University, MUHC-Montreal General Hospital, Room D6 237.F, Room E.16.124.1, 1650 Cedar Avenue, Montreal, QC H3G 1A4 Canada; 5 School of Human Nutrition, McGill University, 21111 Lakeshore Drive, Sainte-Anne-de-Bellevue, QC H9X 2E5 Canada; 6 Department of Medicine, Meakins-Christie Laboratories, McGill University, Montreal, QC Canada

**Keywords:** Frailty, Exercise, Protein, Leucine, Resistance training, Muscle protein synthesis

## Abstract

Frailty, often linked to sarcopenia, involves reduced muscle strength and mass. While sarcopenia has multiple causes, impaired muscle protein synthesis may contribute. Leucine and resistance training (RT) are anabolic stimuli, but the long-term effects of leucine combined with RT in pre/frail older women remain unclear. This double-blind, placebo-controlled study aimed to assess their impact on muscle anabolism and characteristics in women consuming adequate protein. Pre/frail elderly women (*n* = 19, 77.5 ± 1.3 y, BMI: 25.1 ± 0.9 kg/m^2^), underwent 12 weeks of RT with protein-optimized diet (1.2 g**⋅**kg BW^−1^**⋅**d^−1^) and were randomized to 7.5 g/d of leucine (Leu) or placebo 5.1 g/d alanine (Ala). The primary outcome was myofibrillar fractional synthesis rate (MyoFSR) in the postabsorptive and postprandial states. Secondary outcomes are changes in protein content and expression, 1-repetition maximum, body composition (DXA), and myofiber profiles were investigated. Overall MyoFSR increased by 47%, with a concomitant increase in type 1 and 2a myofiber cross sectional area (CSA) (16% and 28%, respectively), and total lean mass (2%). Total AKT, S6, and AMPKα levels were unchanged. However, AKT and S6 phosphorylation increased postprandially before RT, with no change following RT. The number of Frailty Criteria was reduced by 64%, with improvements in physical function and strength. Leucine had no added anabolic benefit. RT with optimal protein intake significantly improved the Frailty Phenotype and increased basal MyoFSR along with type 1 and 2a myofiber CSA and total lean mass, with no added benefit of leucine supplementation.

**Supplementary Information:**

The online version contains supplementary material available at 10.1007/s11357-025-01877-2.

## Introduction

Older adults currently represent 19% of the Canadian population, a percentage that is expected to double in the next 40 years and to triple in those greater than 80 years [1]. With the aging population, there is a consequent increase in the incidence of age-related conditions such as frailty and sarcopenia. Low muscle mass and strength together define sarcopenia as per the recent the Global Leadership Initiative on Sarcopenia (GLIS), which can impact physical performance [2]. Frailty usually manifests by low muscle mass and strength leading to a lowered quality of life. On average women outlive men [3], therefore there is a greater prevalence of frailty in older women [4]. The consequences of frailty include, but are not limited to, increased risk of falls, loss of independence, disability, depression & social isolation, and increased risk of morbidity and mortality [5].

Muscle mass is determined by the net balance between rates of muscle protein synthesis (MPS) and breakdown. Anabolism (muscle accretion) occurs when protein synthesis exceeds protein breakdown while the reverse is true for catabolism. Over the course of a day, the postprandial positive net muscle protein balance counterbalances that of the negative postabsorptive state resulting in an overall maintenance of muscle mass. Current evidence strongly suggests that MPS is a highly regulated process which sensitivity to anabolic stimuli is more susceptible to changes with aging [6], and thus can have a substantial impact on net protein balance.

Aging appears to create a state of protein anabolic resistance with greatest effects via a blunted postprandial anabolic response [7], that can be partially overcome with higher quantities of ingested protein [8]. Accordingly, there is mounting evidence supporting higher dietary protein requirements of older persons than the current recommended dietary allowance of 0.8 g/kg body weight/day [9], and may be as high as 1.2 g/kg body weight/day [10].

The essential amino acid, leucine, is a branched chain amino acid that is not only a substrate for MPS, but also potently stimulates protein synthesis through activation of the mammalian target of rapamycin complex 1 (mTORC1), independently of a rise in insulin [11]. There is strong evidence that chronic leucine supplementation is beneficial in elevating the synthesis of myofibrillar protein [12], however, the effects of leucine supplementation on muscle mass and strength have yielded conflicting results [13].

Resistance training (RT) is another anabolic stimulus that may also decrease during aging. Older adults can increase their muscle strength through RT; however, the muscle hypertrophic (anabolic) response is not comparable to that of a younger population [14, 15]. Although RT has been shown to increase the rate of MPS in older adults [16], a blunted anabolic response to a resistance exercise has been reported [8]. Women appeared to have an attenuated hypertrophic response to training compared to men [17, 18]. Other aspects of sexual dimorphism with respect to muscle fiber profiles have been observed, such as greater type 2 myofiber atrophy with aging in women [19].

The novelty of our approach was to investigate the anabolic effects of RT and chronic leucine supplementation vs. an iso-nitrogenous control, while maintaining sufficient protein intake (~ 1.2 g/kg body weight/day) in older women. We hypothesized that leucine supplementation would enhance both basal and postprandial rates of skeletal MPS leading to greater increases in lean body mass, myofiber cross-sectional area, muscle strength, and function compared to optimized protein intake and RT alone. Thus, our objective was to study in a randomized double-blinded placebo-controlled clinical trial the effects of leucine supplementation while consuming an optimal amount of dietary protein during progressive RT on the rates of skeletal MPS, fiber type composition, protein content and expression, muscle mass, and muscle strength in pre-frail and frail older women.

## Methods

The study was conducted as a registered randomized double-blinded placebo-controlled trial (ClinicalTrials.gov ID: NCT01922167) and was approved and monitored by the McGill University Health Centre (MUHC) Human Research Ethics Board (REB code: 13–211-BMB). Study design and participants profiles have been previously published [20]. All participants read and signed an informed consent form before participating in the study and screening. All participants underwent a 12-week high-intensity progressive resistance exercise training program and followed a protein-optimized diet (~ 1.2 g/kg body weight/day). Half were randomized to receive leucine (2.5 g, 3 times per day) supplementation and the other half an isonitrogenous amount of alanine, an amino acid known not to stimulate muscle protein synthesis independently of insulin (1.7 g, 3 times per day) [21]. All tests were performed before and after the intervention. The muscle protein kinetic studies were performed at the Centre for Innovative Medicine of the MUHC in the postabsorptive (fasting) and postprandial (standard meal) states using primed, continuous infusion of L-[ring-^2^H_5_]phenylalanine.

### Participants & recruitment

Frail or pre-frail community-dwelling elderly women (> 65 y) according to a modified Fried [4] criteria were recruited. Three hundred and four women were screened via telephone, 24 entered the study, and 19 completed the study. Of the 5 participants who left the study, 2 became ill with conditions unrelated to the study, 1 sustained an injury unrelated to the study, 1 moved out of province, and 1 was unable to maintain adherence to the protocol. The remaining 19 participants adhered at least 80% to both the exercise program and supplement intake. Inclusion and exclusion criteria were previously described [20].

### Resistance training

Participants performed resistance exercise three times per week on non-consecutive days, as previously described [20]. Exercises were horizontal leg press, chest press, knee extension, and lateral pulldown. Participants performed 3 sets of 8–15 repetitions for each exercise and resistance was increased by 1–5 lbs (0.45–2.27 kg) when the participant could perform up to 15 repetitions with proper technique. The duration of each set (Time Under Tension, TUT) was obtained (> 35 s) to ensure that the participants were not using momentum to complete the motions. Resistance was determined to consistently be 60–80% of their 1-repetition maximum. The one-repetition maximum (1RM) tests for both the chest press and leg press were performed using fixed resistance machines (plate-loaded or weight stack) rather than free weights, ensuring greater stability and safety for older participants. Prior to testing, participants completed a standardized warm-up consisting of light aerobic activity followed by submaximal practice sets on each machine to familiarize them with the movement pattern. For the 1RM determination, the load was progressively increased in small increments until the participant could successfully complete one full repetition with proper form and without assistance. Adequate rest periods (2–3 min) were provided between attempts to minimize fatigue. The highest load successfully lifted through the full range of motion was recorded as the 1RM for each exercise.

### Supplementation

Participants were randomized into supplement groups by an independent source, based on random generated numbers. Individual doses of powdered supplements of L-leucine (2.5 g, ProteinCo, QC, Canada) or isonitrogenous amounts of L-alanine (1.7 g, PureBulk® OR, USA) were provided in sterile sealed screw-top 100 mL identical containers. Participants were instructed to consume one complete dose of supplement in 80–100 mL of water or sugarless drink at the onset of each main meal (breakfast, lunch, dinner) for the duration of the intervention. Log sheets were collected every 2 weeks to track compliance.

### Dietary protein intake and activity level

Dietary caloric and protein intake was assessed by an initial screening 24-h food recall and subsequent pre-intervention 3-day food diaries. Dietary intake was analyzed using the Food Processor SQL software (Version 10.11.0, ESHA Research, Salem OR). Participants were given instruction and guidance by a study dietitian on how to maintain an isoenergetic protein dietary intake of 1.2 g/kg body weight/day by making minor adjustments to their normal food intake. Food recalls (24 h) were obtained from participants pre-, post-, and at least once at mid-intervention to verify the maintenance of dietary intake. All participants had to wear an accelerometer (ActiGraph GT3X +, ActiGraph, LLC, USA) for three consecutive days (2 weekdays and 1 weekend) before and after the intervention to measure their activity level.

### Outcome Measures

#### Frailty Phenotype

Frailty status was assessed using the modified Fried Criteria [4] at baseline (mandatory for participating) and after intervention. Participants meeting 1–2 of the criteria were categorized as pre-frail, whereas those meeting ≥ 3 criteria, as frail. The five criteria are summed as follows: (1) slowness, identified as a 4-m gait speed of ≤ 1 m/s [22]; (2) weakness, identified as a handgrip strength ≤ 20 kg using a Jamar hydraulic hand dynamometer (Sammons Preston, Inc., IL, USA) [23]; (3) sedentariness, identified by a CHAMPS Physical Activity Questionnaire score ≤ 125 [24]; (4) low muscle mass, identified by a muscle mass index (MMI) < 6.76 kg/m^2^ using BIA (RJL Systems Inc., MI, USA) with the Roubenoff, Baumgartner [25] equation validated for older women; and (5) exhaustion, identified by at least one positive response to either of the 2 following questions: “How often do you feel like ‘I just could not get going’; and ‘Everything I did was an effort’” [4].

#### Body composition

Total weight, BMI, lean body mass (LBM), percent body fat (%fat), and appendicular lean mass index (ALMI) were obtained using a 3-compartment model via dual-energy x-ray absorptiometry (DXA) (GE Lunar iDXA).

#### Physical function & strength tests

All physical function and strength tests were performed on-site at the gym by trained kinesiologists, blinded to the supplement intervention using standard methods. Tests were: 1) Short Physical Performance Battery (SPPB) [26], 2) Timed up-and-go test (TUG) [27], 3)Handgrip strength (Maximal dominant hand) [23], 4) Senior Fitness Test (SFT) [28], 6) One repetition maximum of the four major exercises (leg press, chest press, knee extension, lateral pull-down) [29].

#### Muscle protein tracer studies

**Fig. 1:**
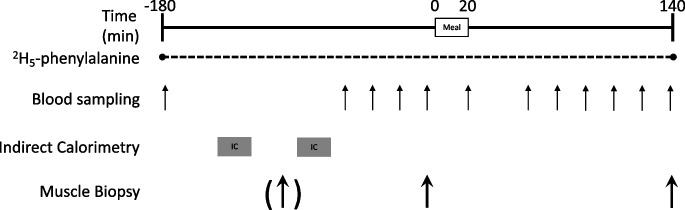
Schematic of the infusion (tracer) study design

Stable isotope tracer studies were performed at least 48 h after completion of the physical function tests pre-intervention, and ~ 48 h post-intervention. Participants arrived at the CIM in the fasting state. Following catheter insertions for tracer infusion and arterialized blood sampling, the fractional rate of MPS was measured in both the postabsorptive and postprandial states using primed (2 μmol/kg) followed by continuous (0.05 μmol/kg/min) infusion of L-[ring-^2^H_5_]phenylalanine (Cambridge Isotope Laboratories, Inc., Andover, MA) tracer (Fig. 1).

#### Pre-intervention

Because participants were naïve of the tracer, the single biopsy approach was taken in the postabsorptive phase, with the collection three hours after the start of tracer infusion. Twenty minutes later, participants consumed a meal replacement, Ensure® (0.52 g leucine per 100 mL), calculated to meet one-third of their daily energy and protein needs (0.67 g protein per kg lean body mass) with added leucine or alanine powder as per group assignment. An oral tracer was added to the meal to maintain isotopic enrichment (6%). A postprandial biopsy was performed 2 h post-meal.

#### Post-intervention

The first biopsy was taken at 1.5 h into the infusion to ensure a linear tracer incorporation, followed by a second biopsy before the meal and a third biopsy 2 h post-meal.

Muscle samples were taken from the vastus lateralis, 20 cm above the knee and 4–5 cm apart, using a UHC needle biopsy (Millennium Surgical Corp.). Fat was removed, and samples were sectioned for various analyses. For MPS analysis, 30 mg of tissue was frozen in liquid nitrogen and stored at −80 °C. For fiber typing, 30 mg transverse sections were mounted on cork, snap-frozen in isopentane cooled by liquid nitrogen, and stored at −80 °C. Some measurements were done on a subset of muscle specimens due to technical issues. Sample sizes for each measurement are detailed in the results section.

#### Measurements of muscle protein synthesis (MPS)

MPS was measured based on the myofibrillar fractional synthesis rate (MyoFSR), calculated according to a precursor-product relationship [30]. Approximately 30 mg of muscle was homogenized and myofibrillar proteins were separated by centrifugation, then the myofibrillar fraction was precipitated and amino acids liberated. The tracer/tracee ratio of phenylalanine was determined by liquid chromatography-tandem mass spectrometry (LC/MS/MS) (Agilent 1290 LC system with 6460 Triple Quadrupole MS). Detection and quantification of phenylalanine was performed using multiple reaction monitoring in positive ion mode with the following transitions: m/z 166 > 120 (phenylalanine); m/z 171 > 125 ([^2^H_5_] phenylalanine). 3-Nitro-L-tyrosine served as an internal standard used to monitor data quality and reproducibility for LC/MS/MS analysis. FSR was calculated according to the precursor-product relationship from rates of [^2^H_5_] phenylalanine incorporation over time: FSR = (E_pb2_ – E_pb1_)/(E_FAA_ ⋅Δ*t*). E_pb1_ and E_pb2_ are enrichment of protein-bound phenylalanine at postabsorptive and 2 h postprandial, respectively; E_FAA_ is the enrichment in the precursor plasma free amino acid pool, and Δ*t* is the length of time between E_pb1_ and E_pb2_. Postabsorptive FSR was calculated from the single biopsy approach in tracer naïve participants, pre-intervention [31].

#### Immunoblotting

Approximately 20 mg of muscle was homogenized in ice-cold lysis buffer using a Mini-bead beater. Muscle homogenates were centrifuged, and supernatants were collected. Protein content was measured using the Bradford assay, and aliquots were mixed with Laemmli buffer and denatured. Protein extracts (20 µg per lane) were separated by SDS-PAGE and transferred onto PVDF membranes. Membranes were blocked and incubated with primary antibodies overnight at 4 °C. The antibodies used targeted total and phosphorylated AKT, S6, and AMPKα, indicating protein synthesis and glycolysis activation. After washing, membranes were incubated with HRP-conjugated secondary antibody and immunoreactivity was detected using enhanced chemiluminescence. Optical densities (OD) of protein bands were quantified using ImageLab software and normalized to loading controls. For each participant, 4 muscle samples (2 pre-intervention and 2 post intervention) were analyzed, and protein OD was expressed as fold change from the pre-intervention postabsorptive sample.

#### Fiber type profiles

Transverse muscle sections were cut on a cryostat at −24 °C, mounted on frosted glass slides, and stored at −80 °C. Slides were thawed and dried at room temperature for 1 h prior to staining. Sections were washed in PBS, blocked with 10% normal goat serum in PBS, and incubated for 1 h at room temperature with antibodies for the following myosin heavy chain (MHC) isoforms: MHC types 1 (BA-F8), 2a (Sc71), and 2x (6H1), and anti-Laminin. Laminin staining delineated fiber margins. After washing, sections were incubated with secondary antibodies Alexa Fluor-350, −594, and −488, then mounted using Prolong Gold Hard Set Mounting Medium. Images were captured with an Axio Imager M2 fluorescence microscope and analyzed using ImageJ software for myofiber cross-sectional area (CSA, μm^2^). Fibers were measured and typed by color, with an average of 215 fibers assessed per muscle section.

#### Statistical analysis

Unless otherwise indicated, data are presented as means ± SEM. Normality was determined using the Shapiro–Wilk test and data non-normally distributed were log transformed. Outliers were determined using the ROUT method. A two-way ANOVA with Tukey’s post hoc was used to compare changes in fiber area for each fiber type between the supplement (group) or training (time) effects. A two-factor repeated measures ANOVA was used to determine the leucine supplementation (group) and exercise training (time) effects for all other outcomes except MyoFSR. When significant interaction effects were observed, post hoc comparisons were performed using the Sidak test. Differences in MyoFSR were determined using a 2 × 2x2 3-way mixed ANOVA with meal (post-absorptive vs. post-prandial) and time (pre- vs. post-RT) as within-subject factors and group (Leu vs. Ala) as the between-subject factor. The sample size estimation was based on a difference of 20% in postprandial MyoFSR between leucine versus alanine placebo groups, with a standard deviation of ~ 15% [12]. Therefore, with an effect size of 1.25, 9 participants per group were required (α = 0.05; β = 0.80). Significance was set at α ≥ 0.05. The 3-way mixed ANOVA was analyzed using IBM SPSS Statistics 24.0 (International Machines Business Corp., Armonk, NY, USA). All other analyses were performed using Prism, version 7.0a (GraphPad Software, Inc., La Jolla, CA, USA).

## Results

### Baseline characteristics

Participant characteristics including age, weight, BMI, frailty status, dietary intake parameters, body composition parameters, MyoFSR, muscle strength, and physical function parameters at baseline were not different between the groups (Tables 1, 2, 3, 4 and 5, Supplementary Table 6). All participants demonstrated high adherence to the exercise sessions, with an overall attendance rate of 90.8%.

**Table 1:** Participant Characteristics of Pre/Frail Women by Supplement Group at Baseline

Characteristic	Ala	Leu
n	9	10
Age (y)	76.2 ± 1.8	78.7 ± 2.1
Weight (kg)	61.8 ± 2.5	62.9 ± 2.9
BMI (kg/m^2^)	23.8 ± 1	26.2 ± 1.3

Data are means ± SEM. BMI: body mass index

**Table 2:** Basal and fed MyoFSR in pre/frail women with and without leucine supplementation before and after 12 weeks of resistance exercise training

	Pre		Post				
Group	Basal	Fed	Basal	Fed	Time effect (p-value)	Meal effect (p-value)	Supplement effect (p-value)
Ala	0.025 ± 0.003	0.053 ± 0.004	0.042 ± 0.007	0.045 ± 0.005^#^	0.065	< 0.001	0.983
Leu	0.028 ± 0.002	0.051 ± 0.003	0.037 ± 0.005	0.050 ± 0.005^#^			

Data are means ± SEM. Ala: *n* = *8*, Leu: *n* = *10*. # depicts a meal x training interaction (*p* < 0.05)

**Table 3:** Frailty Profiles of pre/frail women with and without leucine supplementation before and after 12 weeks of resistance exercise training

	Ala		Leu			
Criteria	Pre	Post	Pre	Post	p-value	Effect
Number of Criteria met	2.7 ± 0.3	0.7 ± 0.3	2.6 ± 0.3	1.2 ± 0.2	< 0.001	Time
Walking speed (m/s)	1.02 ± 0.04	1.20 ± 0.03	0.99 ± 0.05	1.17 ± 0.06	< 0.001	Time
Handgrip strength (kg)	19.2 ± 1.6	22.6 ± 1.9	22.7 ± 2.0	21.7 ± 1.9	0.01	Interaction
SMI (kg/m^2^) (BIA)	6.96 ± 0.23	9.15 ± 0.24	6.32 ± 0.27	8.89 ± 0.20	< 0.001	Time
CHAMPS score	138 ± 52	379 ± 59	121 ± 41	442 ± 73	< 0.001	Time

Data are means ± SEM. Ala: *n* = *9*, Leu: *n* = *10*. SMI: skeletal muscle mass index; CHAMPS: community healthy activities model program for senior’s questionnaire

**Table 4:** Body composition measurements by DEXA in pre/frail women with and without leucine supplementation before and after 12 weeks of resistance exercise training

	Ala		Leu			
Criteria	Pre	Post	Pre	Post	p-value	Effect
Total body mass (kg)	61.8 ± 2.5	62.7 ± 2.3	62.9 ± 2.9	62.2 ± 2.8	0.1	-
LBM (kg)	38.1 ± 1.3	38.9 ± 1.4	35.2 ± 1.4	35.9 ± 1.5	< 0.001	Time
ALMI (kg/m^2^)	6.4 ± 0.2	6.6 ± 0.3	6.5 ± 0.3	6.5 ± 0.6	> 0.05	-
%Fat	36 ± 2.2	35.5 ± 2.1	41.3 ± 1.5	40 ± 1.7	0.015	Time

Data are means ± SEM. Ala: *n* = *9*, Leu: *n* = *10*. ALMI: appendicular lean mass index; BMI: body mass index; LBM: lean body mass; %fat: percent body fat

**Table 5:** Physical Performance testing results in pre/frail women with and without leucine supplementation before and after 12 weeks of resistance exercise training

	Ala		Leu			
Test	Pre	Post	Pre	Post	p-value	Effect
4-m gait speed (m/s)	1.02 ± 0.04	1.20 ± 0.03	0.99 ± 0.05	1.17 ± 0.06	< 0.001	Time
TUG (s)	10.3 ± 0.6	9.1 ± 0.3	10.6 ± 0.6	8.9 ± 0.4	0.0095	Time
SPPB						
Total score	10.0 ± 0.6	11.4 ± 0.2	9.9 ± 0.3	11.2 ± 0.5	0.001	Time
5 chair stands (s)	13.2 ± 1.0	11.1 ± 0.7	13.1 ± 0.5	10.6 ± 0.6	0.001	Time
8’ walk (s)	2.6 ± 0.2	2.5 ± 0.1	2.4 ± 0.1	2.5 ± 0.2	> 0.05	Time
SFT						
30 chair stands (#)	11.1 ± 0.7	13.6 ± 1.1	11.8 ± 0.4	14.0 ± 0.5	< 0.001	Time
Arm curls (#)	16.0 ± 0.9	19.7 ± 1.4	15.8 ± 1.0	20.0 ± 0.6	< 0.001	Time
Sit-and-reach (in)	0.4 ± 2.0	3.8 ± 1.7	−1.3 ± 1.9	1.0 ± 1.3	< 0.001	Time
Back scratch (in)	1.5 ± 0.7	2.5 ± 0.7	−0.8 ± −1.2	0.1 ± 0.9	0.041	Time
8’ up-and-go (s)	7.4 ± 0.9	6.0 ± 0.4	7.9 ± 0.6	7.0 ± 0.5	0.001	Time
1RMs						
Leg press (kg)	75.4 ± 8.6	99.8 ± 9.8	71.2 ± 6.4	96.6 ± 6.4	< 0.001	Time
Chest press (kg)	24.0 ± 1.4	29.5 ± 2.3	23.1 ± 2.3	28.6 ± 1.8	< 0.001	Time
Knee extension (kg)	40.4 ± 2.3	54.0 ± 4.1	49.9 ± 7.7	59.4 ± 7.3	< 0.001	Time
Lat pull-down (kg)	32.7 ± 1.4	38.1 ± 1.4	33.1 ± 1.8	39.0 ± 2.3	< 0.001	Time

Data are means ± SEM. Ala: *n* = *9*, Leu: *n* = *10*. SFT: seniors fitness test; SPPB: short physical performance battery; TUG: timed up and go; 1RM: 1-repetition maximum

**Fig. 2:**
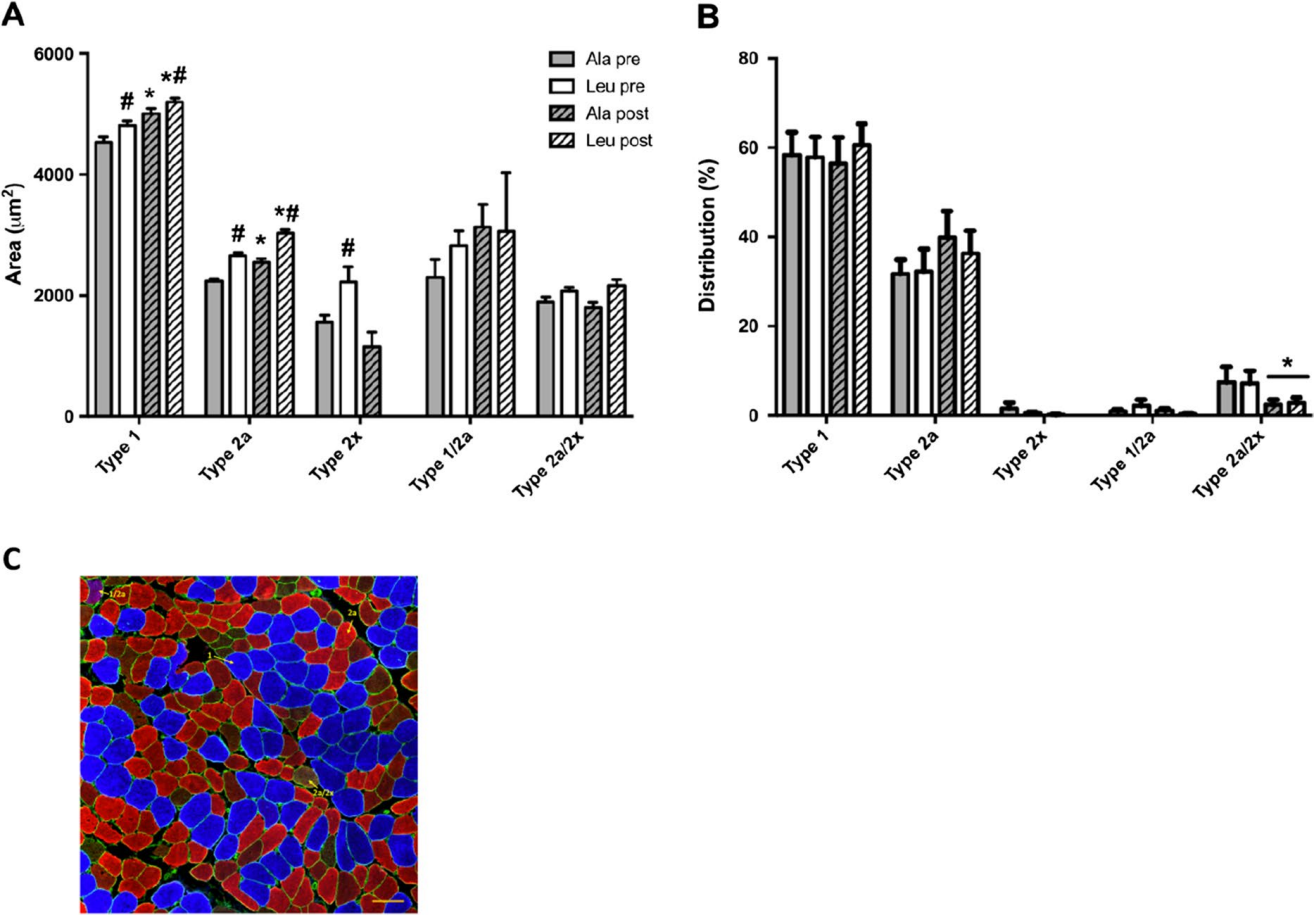
Myofiber cross sectional area (CSA) (**A**), distribution (**B**), and example of immunohistochemically stained section (bar is 100 µm) (**C**), in pre/frail women with and without leucine supplementation before and after 12 weeks of resistance exercise training Data are means ± SEM. Ala: *n=8*, Leu: *n=9*. * Depicts time effect, # depicts Leu different from Ala at same timepoint(*p<0.05*)

Groups did not differ at baseline for average energy intake and for the percentage of time spent in sedentary, light, or moderate activity (Supplementary Table 7). At baseline, the CSA of types 1, 2a, and 2 × fibers were greater in the Leu group than Ala group (*p* = 0.024, < 0.001, and 0.011, respectively) (Fig. 2A). No baseline differences were observed for fiber type distribution (Fig. 2B).

### Fiber size and fiber type proportion

Cross-sectional area of types 1, 2a, and 2 × were greater in the Leu group prior to RT compared to the Ala group (Fig. 2A). Both groups significantly increased the CSA of fiber types 1 and 2a in response to training (time effect, *p* < 0.001), with no interaction effect (Fig. 2A). No significant effects were observed for the CSA of types 1/2a and 2a/2 × fibers (Fig. 2A). No differences at baseline and no significant effects of training were observed for the proportions of types 1, 2a, 2x, or 1/2a. Both groups significantly decreased the proportion of type 2a/2 × fiber post-intervention (time effect, *p* = 0.021) (Fig. 2B), with no group or interaction effects observed.

### Myofibrillar Fractional Synthesis Rate

**Fig. 3:**
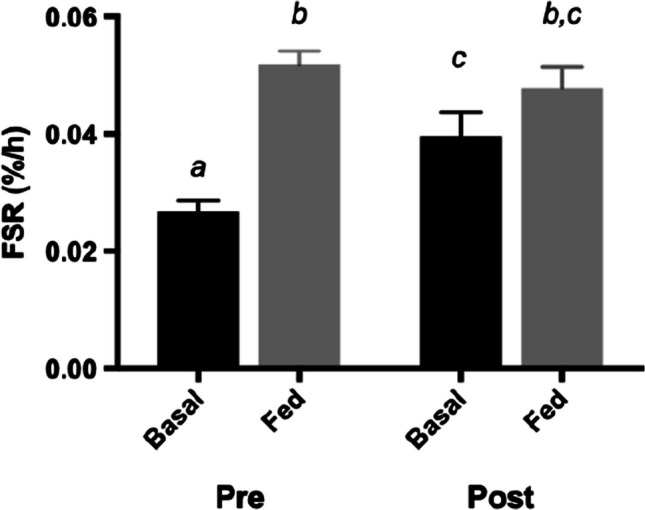
Myofibrillar fractional synthesis rate in pre/frail women in the postabsorptive (basal) and postprandial (fed) state, before and after 12 weeks of resistance exercise training. Data are mean ± SEM. Both groups combined. Bars not sharing the same letter are significantly different (meal × time interaction effect, *p* < 0.05)

Plasma enrichment remained stable throughout the postprandial and increased early postabsorptive states and stabilize (average ± 10% with SD < 1%, data not shown. Ala: *n* = 8, Leu: *n* = 10). A meal effect on MyoFSR was observed (*p* < 0.001) (Table 2). Feeding significantly increased MyoFSR at baseline and after the intervention. The training intervention significantly increased MyoFSR overall, but no significant meal x training interaction effect was observed (Fig. 3). Analysis of the interaction revealed that the basal MyoFSR, was significantly increased post-intervention (+ 47%) but not the meal MyoFSR has been observed.

### Protein phosphorylation

**Fig. 4:**
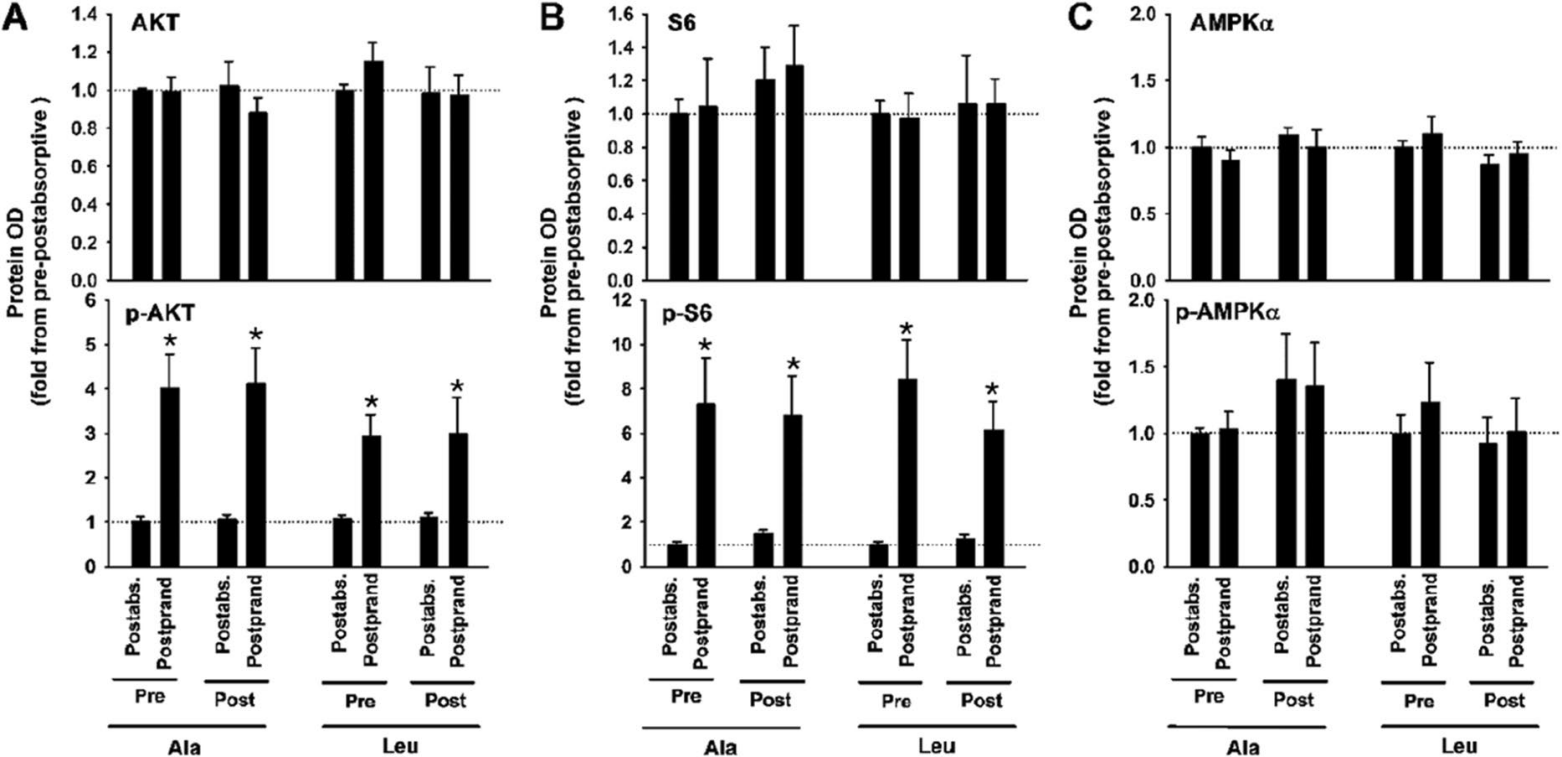
Total and phosphorylated AKT, S6 and AMPK proteins in pre/frail women in the postabsorptive and postprandial (fed) states, before and after 12 weeks of resistance exercise training in the Ala and Leu groups. Data are mean ± SEM. Ala: *n* = 8, Leu *n* = 9. Pre: prior to exercise resistance training. Post: post resistance exercise training. *p < 0.05 compared to the postabsorptive state

Figure 4 illustrates changes in total and phosphorylated levels of AKT, S6, and AMPKα proteins in muscle samples from the Ala and Leu groups. Samples were analyzed for each participant in the postabsorptive and postprandial states, before and after the intervention using western blotting. No changes were observed in total AKT, S6, or AMPKα levels between the postabsorptive and postprandial states, both pre- and post-intervention. However, phosphorylation of AKT and S6 significantly increased in the postprandial state compared to the postabsorptive state before RT, and this increase was not altered by RT. AMPKα phosphorylation showed no difference between the postabsorptive and postprandial states at both pre- and post-intervention in either group.

### Frailty Criteria

Both groups significantly decreased their number of frailty criteria following 12 weeks of RT (Table 3), and no group or interaction effects were observed. The percentage of participants with a reduced Frailty score by 1, 2, or 3 criteria was: 5.3%, 21.1%, and 15.8% in Ala group; 26.3%, 15.8%, and 15.8% in Leu group. Both groups significantly increased their CHAMPS score (time effect, *p* = 0.001) (Table 3), with no supplement or interaction effects observed. Both groups significantly increased their SMI (time effect, *p* = 0.001) (Table 3), with no group or interaction effects. The Ala group increased their handgrip strength (*p* = 0.010) post-intervention (interaction effect, *p* = 0.01) (Table 3), but no time or interaction effects were observed.

### Body Composition

No significant effects were observed for total body mass (Table 4). Both groups increased their LBM (time effect, *p* < 0.001) and decreased their %fat (time effect, *p* < 0.015) with no group or interaction effects (Table 3). ALMI did not change after the intervention and no significant difference were observed between groups.

### Physical Function & Strength Tests

Strength for each of the four 1RM exercises (leg press, chest press, knee extension, lateral pull-down) significantly increased in both groups (time effect, *p* < 0.0001) (Table 5). Both groups significantly increased their 4-m gait speed (time effect, *p* < 0.0001), SPPB score (time effect, *p* = 0.002), number of chair stands completed in 30 s (SFT: time effect, *p* = 0.0006), number of arm curls completed in 30 s (SFT: time effect, *p* < 0.001), upper body flexibility (SFT, back scratch: time effect, *p* = 0.042), and lower body flexibility (SFT, sit-and-reach: time effect, *p* < 0.0001) (Table 5). Both groups significantly decreased their TUG time (time effect, *p* = 0.010), time to complete 5 chair stands (SPPB: time effect, *p* = 0.0001), and 8’ up-and-go time (SFT: time effect, *p* = 0.001) (Table 5). No group effect or interaction were observed in all tests described above.

### Dietary Intake

No significant effects were observed between any timepoint (pre-, mid-, or post-intervention) for energy intake, absolute or relative protein intake (Supplementary material).

### Accelerometer

No significant effects were observed for average physical activity energy expenditure, percentage of time spent in sedentary, light, or vigorous activity (Supplementary material, Ala: *n* = 7, Leu: *n* = 8). A group effect was observed for percentage of time spent in moderate activity (*p* = 0.019), with Ala higher than Leu at both time points.

## Discussion

We investigated the impact of leucine supplementation combined with resistance training (RT) in pre-frail and frail older women receiving optimized dietary protein intake. We hypothesized that 12 weeks of RT, with leucine supplementation, would enhance postprandial muscle protein synthesis (MPS), leading to greater increases in lean body mass (LBM), myofiber cross-sectional area (CSA), muscle strength, and physical function compared to RT alone.

The main findings were that leucine supplementation did not significantly enhance the effects of RT. Therefore, results for RT were analyzed with leucine and alanine-supplemented participants combined.

First, we observed that basal (postabsorptive) myofibrillar fractional synthesis rate (MyoFSR) were significantly higher (47%) post-intervention, with no change in postprandial rates. A study in healthy older adults (~ 71 years) showed similar results reporting that basal mixed protein FSR significantly increased by ~ 30% after 12 weeks of RT without further increasing the MPS response to feeding [32]. Our results agree with the aforementioned study, that our RT-induced increase in basal MyoFSR was ~ 1.5-fold. A possible reason why we observed a dramatically higher response is that we investigated the myofibrillar fraction, while the former study investigated mixed protein FSR. A 2001 study [33] supports our findings, in which FSR was measured in isolated myosin heavy chains (MHC) before and after 3 months of RT in 46–79 year-old men and women. Authors found that the MHC FSR increased by 47% post-RT in the exercise group only. Our observations result from a more specific cohort of aged individuals in whom we found that even in the context of frailty, older women can respond to RT. We investigated MyoFSR over the two nutritional states (postabsorptive and postprandial) in a fraction consisting of both major functional contractile proteins (myosin and actin), and thus we report a more comprehensive analysis than the aforementioned study. Of note, no significant improvement was observed before and after the intervention in the postprandial state. This might be due to the strong stimuli of meal (energy and protein ingested) that masks the contribution of exercise. This absence of postprandial enhancement with leucine may also reflect the optimized protein content of the test meal, which likely achieved a maximal anabolic response and left limited scope for further stimulation by leucine supplementation.

We observed no change in myofiber distribution except for a significant decrease in type 2a/2 × fibers post-intervention, likely due to the 2 × to 2a shift seen with RT [32]. Our findings align with previous studies showing RT does not affect type 1 fiber distribution in older adults, including older women. Some studies [22], but not all [32], reported an increase in type 2a fibers with RT in older adults. Our results align with the latter, showing no change in type 2a distribution post-intervention.

Participants significantly increased their type 1 and 2a fiber CSA, the first known study to show an increase in type 1 myofiber CSA after 3 months of RT in frail and pre-frail older women. Previous RT interventions reported no significant gains in type 1 myofiber CSA in older adults [19], unlike our finding of a 16% mean increase. Consistent with the increased MyoFSR and in concert with a significant increase in type 1 and 2a myofiber CSA is the whole LBM gain of an average of 750 g (2%) in both groups. However, no changes in ALMI were observed, which might be due to insufficient statistical power, as changes in ALMI tend to be smaller to that of LBM and thus require a larger sample size to detect statistical differences. Total and phosphorylated levels of AKT, S6, and AMPKα did not change when compared to pre-intervention levels in both postprandial and postabsorptive states. Some studies have reported earlier changes, observing improvements in phosphorylated proteins as soon as one hour after meal ingestion [34]. This difference in timing may explain why our muscle biopsies performed two hours after ingestion of the meal did not detect any changes following the intervention.

We reported the favorable change in Frailty Phenotype with RT in older women with an optimized dietary protein intake. In addition to the fundamental changes observed in MPS and muscle fibers, from a clinical perspective, 9 participants improved from frail to pre-frail, 4 from pre-frail to healthy, and 2 from frail to healthy categorization. Only 3 pre-frail participants did not improve upon their category. These changes were observed following the significant increase in lean muscle mass and strength and an improvement in functional capacity.

The lack of benefit of leucine supplementation raises the possibility that perhaps the 2.5 g dose of leucine per meal was not high enough to further stimulate MPS. Future studies are required to determine the optimal dose of leucine in the context of a mixed-macronutrient meal needed to effectively enhance MPS in older persons. Additionally, another possible explanation is that the protein content of the meal was optimized to maximize muscle protein synthesis (MPS) in this population. Previous studies have shown that 0.61 g of protein/kg of LBM is required to achieve maximal MPS in males, and since we were providing 0.68 g of protein/kg LBM in the test meal to our older frail women, they have reached their maximal threshold [8]. Another possible reason why we did not observe an enhanced effect of leucine supplementation in our study is that perhaps the RT in combination with sufficient dietary protein intake was an optimal anabolic stimulus, resulting in no anabolic deficits for leucine to improve upon in this cohort. It is known that the anabolic effect of RT is elevated when combined with protein, and several systematic reviews reported compelling findings that anabolic resistance seen with aging can be overcome with RT and sufficient provision of protein [6].

Strengths of the current study include that our cohort ‘diet (through multiple food-recalls) and physical activity (by accelerometry) were controlled and taken into consideration into statistical analysis. Previous studies have usually neglected to control for these potent confounding factors. In the same regard, our study also utilized an appropriate placebo (alanine), which provided nitrogen equivalent to leucine. An important limitation of our study is that muscle protein breakdown was not measured, which is necessary to obtain a true measure of net protein balance. In addition, muscle mass was not measured and techniques such as MRI or D3 creatine should be considered to estimate with more precision. Future studies should include institutionalized/hospitalized frail and also sarcopenic persons. Because one can be frail without significant muscle atrophy, studying truly sarcopenic persons would provide information relating directly to states of muscle atrophy.

In conclusion, leucine supplementation at 2.5 g per meal did not provide additional benefits beyond 12 weeks of resistance training in pre-frail and frail older women habitually consuming an optimal amount of dietary protein. The resistance training intervention alone significantly improved the Frailty Phenotype, enhanced physical function and strength, mediated through increased basal myofibrillar protein synthesis rates alongside gain in type I and type IIa myofiber cross-sectional area and whole-body lean mass. These results demonstrate that resistance training combined with optimized protein intake constitutes a robust and effective anabolic strategy in this population. Clinically, ensuring an intake of approximately 1.2 g/kg/day of high-quality protein containing all essential amino acids may maximize muscle protein synthesis and functional outcomes. This approach provides health professionals with practical guidance to design interventions that can facilitate the transition from frailty to prefrailty, or potentially reverse frailty altogether, thereby improving mobility, independence, and quality of life in older adults.

## Supplementary Information

Below is the link to the electronic supplementary material.

Supplementary file 1 (DOCX 34.8 KB)
