# Supplementary material for: Resistance training, but not leucine, increased basal muscle protein synthesis and reversed frailty in older women consuming optimized protein intake
Source: GeroScience. 2025 Sep 9;48(3):3971–82. doi: 10.1007/s11357-025-01877-2 (PMC13356255; doi:10.1007/s11357-025-01877-2)
Supplement: Supplementary file 1 — (DOCX 34.8 KB) [file 11357_2025_1877_MOESM1_ESM.docx]

Supplementary material

|  |  | Ala | |  | Leu | |  |  |
| --- | --- | --- | --- | --- | --- | --- | --- | --- |
| Criteria | Pre | Mid | Post | Pre | Mid | Post | p-value | Effect |
| Energy Intake (kcal/d) | 1680 ± 99 | 1513 ± 81 | 1574 ± 86 | 1555 ± 89 | 1546 ± 115 | 1696 ± 89 | >0.05 | - |
| Protein intake (g/d) | 76.6 ± 2.5 | 76.9 ± 6.5 | 76.4 ± 4.2 | 74.6 ± 3.0 | 76.6 ± 3.3 | 74.1 ± 4.4 | >0.05 | - |
| Protein intake (g/kg/d) | 1.24 ± 0.06 | - | 1.23 ± 0.09 | 1.22 ± 0.07 | - | 1.21 ± 0.09 | >0.05 | - |
| Leucine Intake (g/d) | 5.77 ± 0.20 | 4.65 ± 0.22 | 5.52 ± 0.32 | 5.10 ± 0.30 | 5.16 ± 0.24 | 5.39 ± 0.37 | 0.013 | Interaction |

Table S1. Caloric, protein and leucine habitual dietary intake in pre/frail women with and without leucine supplementation before and after 12 weeks of resistance exercise training

Note: protein and leucine values do not include supplement intake

Data are means ± SEM. Ala: *n=9*, Leu: *n=10*

Table S2. Habitual Physical Activity Profile Measured by Accelerometry in pre/frail women with and without leucine supplementation before and after 12 weeks of resistance exercise training

|  | Ala | | Leu | |  |  |
| --- | --- | --- | --- | --- | --- | --- |
|  | Pre | Post | Pre | Post | p-value | Effect |
| Average kcal | 341 ± 58 | 363 ± 52 | 290 ± 40 | 272 ± 37 | >0.05 | - |
| %Sedentary | 87.4 ± 1.7 | 87.9 ± 1.1 | 88.4 ± 1.3 | 89.6 ± 1.2 | >0.05 | - |
| %Light | 10.7 ± 1.5 | 9.6 ± 0.9 | 10.4 ± 1.0 | 9.0 ± 1.0 | >0.05 | - |
| %Moderate | 1.8 ± 0.4 | 2.3 ± 0.3 | 1.0 ± 0.3 | 1.2 ± 0.3 | 0.019 | Group |
| %Vigorous | 0.2 ± 0.1 | 0.2 ± 0.0 | 0.1 ± 0.0 | 0.2 ± 0.1 | 0.040 | Baseline |

Data are means ± SEM. Ala: *n=7*, Leu: *n*=8.
